# Burden of Disease, Early Diagnosis, and Treatment of Merkel Cell Carcinoma in Latin America

**DOI:** 10.1200/JGO.18.00041

**Published:** 2018-06-04

**Authors:** Rafael A. Schmerling, Jose G. Casas, Gabriela Cinat, Fabio Ernesto Grosso Ospina, Luiza E.B.P. Kassuga, Jorge Luis Martinez Tlahuel, Luis Daniel Mazzuoccolo

**Affiliations:** **Rafael A. Schmerling**, Beneficiência Portugesa de São Paulo, São Paulo; **Luiza E.B.P. Kassuga**, National Cancer Institute, Rio de Janeiro, Brazil; **Jose G. Casas**, Hospital Alemán de Buenos Aires; **Gabriela Cinat**, University of Buenos Aires; **Luis Daniel Mazzuoccolo**, Hospital Italiano de Buenos Aires, Buenos Aires, Argentina; **Fabio Ernesto Grosso Ospina**, Centro Nacional de Oncología de Colombia, Bogotá, Colombia; and **Jorge Luis Martinez Tlahuel**, National Cancer Institute, Mexico City, Mexico.

## INTRODUCTION

Merkel cell carcinoma (MCC), first described by Cyril Toker in 1972,^1^ is a rare and aggressive skin cancer. Although it accounts for less than 1% of malignant skin tumors, it is the second leading cause of death from skin cancer behind melanoma.^2^ Despite its aggressive behavior, MCC may be curable in patients with local and node-positive disease.^3^ Even with a high rate of local and distant recurrence, treatment options exist that can improve overall survival and quality of life. Early diagnosis and timely intervention are key to improving health outcomes. The purpose of this work is to briefly review the features and treatment of MCC so that health care providers and policymakers are familiar with the disease and recognize the current limitations in Latin America that are barriers to improved outcomes.

## EPIDEMIOLOGY

The annual incidence of MCC worldwide varies between 0.13 and 1.6 per 100,000 persons, and it is unclear whether this range reflects different environmental or genetic factors or issues related to case finding.^4-8^ The incidence of MCC seems to be growing. The SEER Program in the United States documents a three-fold increase, adjusted for age, from 0.15 to 0.44 per 100,000 persons from 1986 to 2011^2^; however, here, too, it is unclear whether the increase is a result of improved surveillance or because risk factors for the disease are increasing. If the incidence is truly increasing, several factors may contribute, such as aging of the population, the global increase in UV ray exposure, and a greater number of people who are immunocompromised for several possible reasons.

MCC usually occurs in the elderly, between the seventh and eighth decades of life; only approximately 5% of cases occur in people age younger than 50 years.^9^ MCC is extremely rare in children,^10^ is much more frequent in whites^9^ than in people of other races, and more frequent among men than women.^7^ Patients who are diagnosed with MCC also have an increased likelihood of having other neoplasms^11^; the coexistence of MCC with chronic lymphocytic leukemia is particularly well documented.^12^

Unfortunately, there are no population-based studies or national registries in Latin America that provide data on MCC. Virtually all data published from Latin America comes from case series or individual hospitals; therefore, we do not know whether the incidence of MCC among the countries in Latin American is similar, or even if the incidence differs between areas within a country. Clearly, establishing national registries throughout the region should be a high priority.

## ETIOLOGY AND PATHOPHYSIOLOGY

The etiology of MCC is likely multifactorial. UV ray–induced skin damage, immunosuppression and Merkel cell polyomavirus (MCPyV) infection are thought to be the major risk factors associated with MCC. Nonetheless, the pathogenesis of the disease is poorly understood.^13,14^

The positive association between MCC and UV radiation is well established.^15^ Fair-skinned individuals have a higher incidence of MCC than do those with darker skin.^16^ More than 50% of lesions typically develop on sun-exposed skin, like the head, neck, and arms.^4,17^ MCC is also frequently diagnosed with other tumors that are associated with sun exposure.^11,18^ Other characteristics that support the link between UV radiation and MCC are the higher occurrence in chronically sun-exposed elderly patients and the higher incidence in people who are treated with UVA phototherapy.^16,19^ This is another reminder for health care professionals to reinforce the need for sun exposure protection.

Immunosuppressive conditions, such as lymphoproliferative malignancies, organ transplant, and HIV infection, seem to be important risk factors.^20,21^ MCC develops more frequently and at a much younger age in exposed individuals. Approximately 8% to 10% of MCC cases are related to severe immunosuppression.^22,23^ Data from population-based cancer registries show that patients who undergo organ transplantation had a 24-fold higher risk of developing MCC compared with immunocompetent patients, and this risk increases with time from transplantation.^20-22^ In addition, incidence rates rise steeply with increasing age at transplantation.

In 2008, Feng et al^24^ first reported the existence of MCPyV in MCC tumor specimens. The authors reported high rates of viral DNA and clonal integration of the virus into the tumor genome, which suggested that infection and integration preceded clonal expansion of tumor cells, making MCPyV infection a contributing factor in the pathogenesis of MCC. Despite the high prevalence of MCPyV—the seroprevalence in the US population is approximately 60% to 80%—the incidence of MCC is low. Thus, MCPyV infection is not sufficient for the development of MCC.

Another potential risk factor for MCC is chronic arsenic exposure. The relationship between arsenic exposure and skin and solid tumors has been well documented,^25^ and one study from Taiwan linked arsenic exposure with MCC.^26^ Many people in Latin America live in areas with naturally elevated levels of arsenic in drinking water. It is unclear whether some cases of MCC in the region may result from high arsenic exposure. More studies are needed to clarify this concern.

## DIAGNOSIS

**Fig 1:**
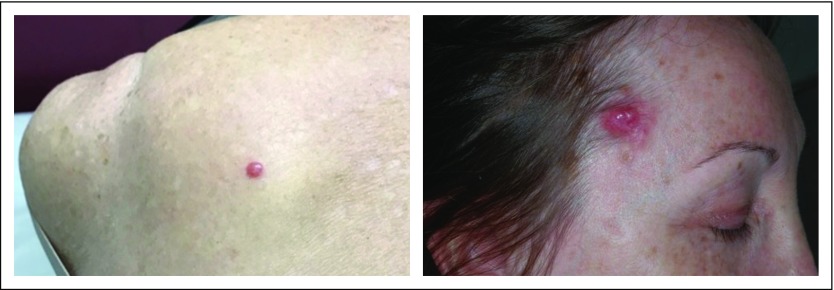
Solitary, painless, and firm, with red-violet or red-blue appearance.

**Fig 2:**
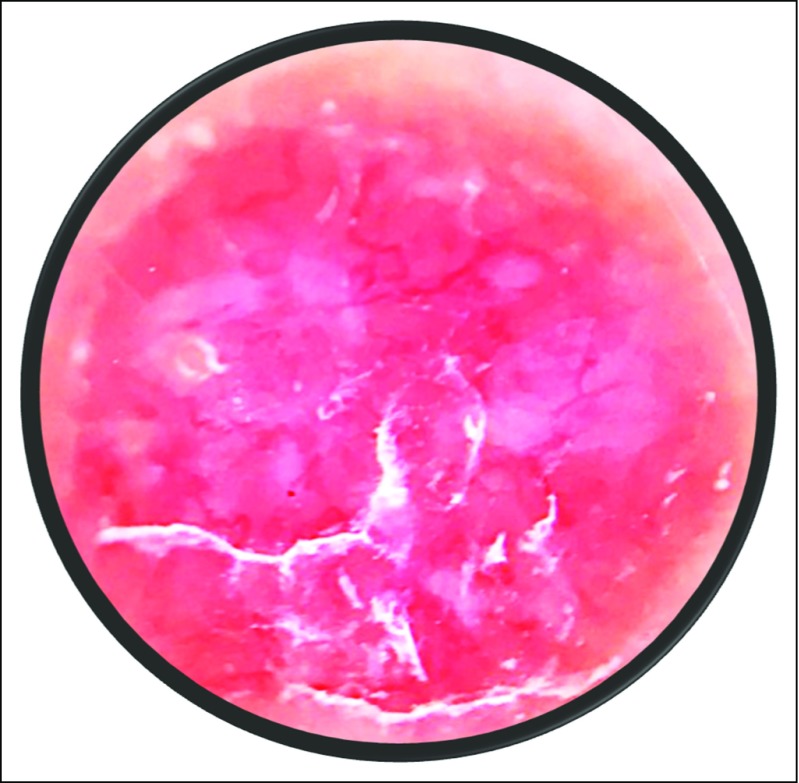
Dermoscopic reveals sparse and irregular linear vessels and milky red areas.

As a result of the low incidence of MCC, it is likely that most physicians will rarely encounter a case, and thus, the disease will likely be overlooked. Dermatologists are the health professionals best able to identify MCC cases. Nonetheless, all physicians should be aware that MCC usually presents as a rapidly growing nodule that is solitary, painless, and firm, and that has a red-violet or red-blue appearance^22^ (Fig 1). Dermoscopic findings are sparse and include irregular linear vessels and milky red areas (Fig 2).^27^ The MCC lesion may be mistaken for benign lesions or other malignancies, such as squamous cell carcinoma, cutaneous lymphoma, or a metastasis from another tumor.^16,28^ As MCC is easily overlooked or misdiagnosed, any skin nodule that has the above characteristics should trigger a high index of suspicion, and the patient should be referred to a dermatologist.

The acronym, AEIOU, can be helpful as a diagnostic tool. It stands for asymptomatic, (rapidly) expanding, immunosuppression, older age (age > 50 years), and UV radiation exposure. The majority of patients with MCC present with three or more of these characteristics.^22^

**Fig 3:**
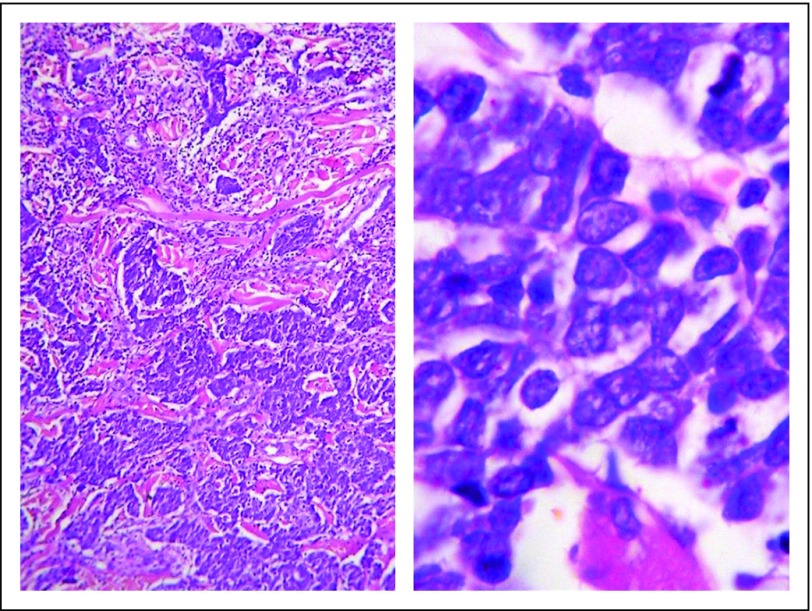
Pathology of Merkel cell carcinoma: proliferation of uniform, small, round, blue undifferentiated cells with spherical or oval nuclei and scant cytoplasm. (Left) Sheets of small cells extending throughout the dermis (hematoxylin and eosin [H&E]). (Right) Small, round, blue undifferentiated cells at a higher magnification (H&E).

**Fig 4:**
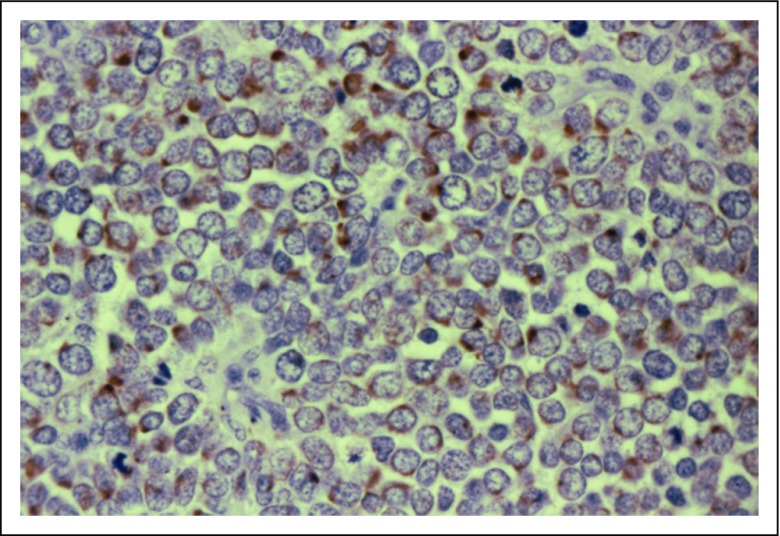
Immunohystochemistry of Merkel cell carcinoma. Dot-like positivity image for cytokeratin 20 (immunoperoxidase stain).

A definitive diagnosis of MCC is made on the basis of histopathology and immunohistochemistry. On hematoxylin and eosin examination, MCC is characterized by a proliferation of uniform, small, round, blue undifferentiated cells with spherical or oval nuclei and scant cytoplasm (Fig 3), high mitotic rate, apoptotic bodies, and occasional necrosis. In clinical practice, the most important immunohistopathologic features are dot-like positive CK-20 (Fig 4) and negative TTF-1 and CK-7 stainings.^29^

## STAGING

MCC is associated with a high risk of relapse and disease-related mortality.^30,31^ It usually spreads first to regional lymph nodes, which makes sentinel lymph node biopsy (SLNB) important for staging.^16^ In 2017, the American Joint Committee on Cancer^32^ defined a new staging system on the basis of data from 9387 patients who were diagnosed between 1998 and 2012 (Tables 1 and 2). The results showed that more than one half of patients (65%) were diagnosed with local disease, with a 5-year survival of 56%. Five-year survival dropped as the depth of infiltration increased, from 56% in T1 disease to 32% in T4 disease. Regional involvement was diagnosed in 26% of patients, with a 5-year survival of 35%. In patients with clinically negative nodal involvement but positive SLNB, the 5-year survival rate was 40%, whereas in patients with clinically positive nodal involvement, survival was 27%. Finally, 5-year survival at diagnosis in patients with distant metastases was approximately 14%, with a mean survival of 6 to 10 months.^16,32-34^

**Table 1:**
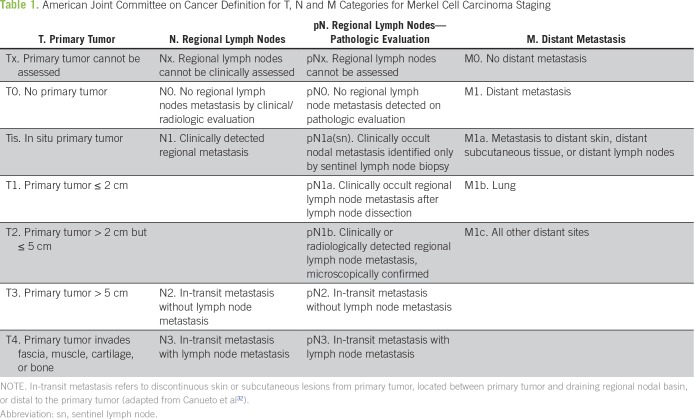
American Joint Committee on Cancer Definition for T, N and M Categories for Merkel Cell Carcinoma Staging

**Table 2:**
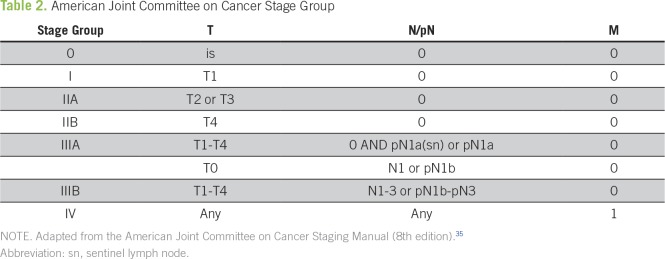
American Joint Committee on Cancer Stage Group

After MCC is confirmed, patients must be assessed to rule out metastatic disease. Whole-body positron emission tomography–computed tomography (PET-CT) is the preferred test, but when unavailable CT scan or magnetic resonance imaging should be used.^36^ One study demonstrated that PET-CT results in staging changes in 33% of patients and management changes in 43% of patients.^37^ Regional lymph node ultrasound is frequently used to assess lymphatic drainage in accessible areas and can have accuracy of up to 90% for distinguishing between benign or malignant nodes.^16,38^

Nodal metastases are found in approximately 30% of patients at the time of diagnosis^28,39^ and in more than 80% of patients over the course of the disease.^40^ A retrospective study of 8,044 patients with MCC demonstrated a risk of nodal involvement of 14% in tumors that were 0.5 cm in size, which increased to 25% in tumors that were 1.7 cm in size, and to 36% in tumors that were > 6 cm^41^; this is why SLNB is recommended for clinically negative lymph nodes. In addition, the number of compromised lymph nodes affects 5-year survival (zero nodes, 76% survival; one node, 50%; two nodes, 47%; three to five nodes, 42%; more than six lymph nodes, 24%).^38^ As in melanoma, SLNB for head and neck MCC is not as accurate for detecting tumor cells as the procedure is for tumors on the extremities and trunk.^37,38^

Considering that surgery may alter lymphatic drainage, SLNB must be performed simultaneously with definitive resection of the primary tumor.^38,42^ The rate of false-negative results has been estimated at 30% and may drop to 22% when immunohistochemical studies are performed.^43,44^

In 10% to 20% of MCC cases, the primary tumor is not found even after thorough workup.^39,45^ Spontaneous regression of the primary tumor might explain this circumstance.^46^

## TREATMENT

Because of the rarity of MCC, there are no randomized controlled trials that have compared different therapeutic approaches. Support for treatment alternatives relies on case series, retrospective and pooled analyses, and, more recently, phase II trials. Recommended treatment depends on the stage of the disease, the location of the tumor, and patient comorbidities.

## SURGERY

For early-stage MCC, wide surgical excision is the treatment of choice for the primary lesion. The main goal is to remove the entire tumor with wide margins, as suboptimal excision is associated with a higher risk of recurrence. National Comprehensive Canter Network (NCCN) and European Association of Dermatology-Oncology/European Organization for Research and Treatment of Cancer guidelines recommend margins of 1 to 2 cm.^34,47^ Mohs micrographic surgery has also been suggested in patients in whom it is difficult to obtain adequate margins, such as on the face.^48^ This procedure should be reserved for select patients only and performed by surgeons who are experienced in this procedure.

Lymph node involvement is common, detected in 30% (range, 15% to 66%) of patients at the time of diagnosis, and in 79% of patients throughout disease progression. Approximately one third of patients with clinically palpable, but radiologically negative, lymph nodes present micrometastasis.^38^ Although survival benefit has not been demonstrated in a randomized controlled trial, the potential for SLNB as a prognostic strategy became obvious in a retrospective comparison between patients with clinically negative lymph nodes compared with those with pathologically negative lymph nodes.^3^

If SLNB is positive, complete dissection of the compromised lymph node basin is the treatment of choice, as it is in clinically positive disease. In those patients who cannot undergo surgery, radiation should be undertaken and the lymph node tumor drainage basin should be irradiated.^38^

Patients with unknown primary MCC restricted to nodal disease are treated with a combination of surgery and radiotherapy. These patients are considered to have a better prognosis than those with MCC with an identifiable primary tumor and nodal involvement.^33,34,46,49^ Radiotherapy may also be considered as primary therapy in patients who are not candidates for surgical treatment.

## RADIOTHERAPY

MCC is a highly radiosensitive tumor, and postoperative radiotherapy is recommended to reduce local recurrence, although there has been no controlled trial to evaluate this treatment modality.^38,50^ Retrospective analyses have produced conflicting results with regard to the survival benefit of radiotherapy^48,51^; however, most of the data suggest that local and locoregional relapse-free survival are improved by adjuvant radiotherapy.^52-55^ A large analysis of this issue was performed using the National Cancer Database.^56^ Nearly one half of patients were treated with surgery followed by radiotherapy. Compared with patients who were treated with surgery alone, those who were treated with combination therapy had significantly better overall survival (stage I: hazard ratio [HR], 0.71; 95% CI, 0.64 to 0.80; *P* < .001; stage II: HR, 0.77; 95% CI, 0.66 to 0.89; *P* < .001). Among patients with stage III disease, there was no difference between the two groups (HR, 0.98; 95% CI, 0.86 to 1.12; *P* = .80).^56^

The NCCN and European Association of Dermatology-Oncology/European Organization for Research and Treatment of Cancer recommend local radiation of the tumor bed after surgery, regardless of stage.^47,48^ Adjuvant radiation of the nodal drainage sites is recommended for patients with positive lymph nodes and for those whose lymph node status is unknown. It may also be considered for those patients with negative lymph nodes who are at high risk of nodal relapse. Radiotherapy alone is considered the treatment of choice in patients with advanced age and many comorbidities that contraindicate surgery.^38^

There are insufficient data to suggest one follow-up schedule is better than another; however, NCCN guidelines^47^ recommended that patients receive follow-up visits with physical examination of the entire skin and lymph nodes every 3 to 6 months for 3 years and every 6 to 12 months thereafter. Imaging studies should also be considered.^47^

## CHEMOTHERAPY

In addition to being radiosensitive, MCC is also a chemosensitive disease in terms of tumor response, although after chemotherapy, benefit in time to progression remains disappointing.^56^ There is also no clear evidence that chemotherapy improves MCC survival. Instead, it is associated with tumor shrinkage and a decline of tumor-related symptoms.^38,49,57^ For many years now, metastatic disease has been treated with platin and etoposide combinations or cyclophosphamide, doxorubicin, or vincristine. In most countries, this is still the standard therapy and, until recently, was the only therapy for metastatic disease. Data do not support the use of adjuvant chemotherapy.^57^

## IMMUNOTHERAPY

Recently, data on the use of a new class of therapeutics, called immuno-oncologic agents, have been accumulating, with response rates between 50% and 73% in patients who have had no prior systemic therapy and 32% in those previously treated.^58-60^ These drugs seem to be associated with a longer duration of response and a longer survival benefit than what has been observed with chemotherapeutic agents, although no head-to-head comparisons have been made. Nonetheless, these newer agents may well become the new standard of treatment.

One such immuno-oncologic agent, the checkpoint inhibitor, avelumab, is a fully humanized monoclonal IgG1 antibody that binds to the programmed death ligand-1.^61,62^ A phase II multicenter trial with avelumab in patients who were previously treated with systemic agents demonstrated an objective response in 32% of patients and 52% were alive at 1 year. This compares with a median survival of 5.7 months in patients with advanced disease who receive second-line chemotherapy.^59^ Serious adverse events related to treatment with avelumab were reported in less than 10% of patients,^59^ which is much lower than that observed with chemotherapeutic agents.^63^ Avelumab has been approved by the European Medicines Evaluation Agency for patients who experience failure with one systemic therapy and by the US Food and Drug Administration, independent of prior therapy.^64,65^ There are many ongoing clinical studies using other immuno-oncologic agents for the treatment of MCC. Thus far, no biomarker or clinical feature has been established as a predictive tool to aid in treatment selection.

## RAISING AWARENESS IN LATIN AMERICA

As noted, accurate information about MCC in Latin America is scarce. There are many possible explanations for the lack of robust data. First, there are significant social, cultural, geographic, and economic differences between and within countries that make data collection difficult and costly. In addition, countrywide registries do not exist or, at best, are in the initial phases of development. Implementation of electronic medical records and centralized storage of information has been increasing in the region, which eventually will generate better data on infrequent tumors, such as MCC. The diversity of ethnicities in the region, along with wide variations in skin color and UV radiation exposure make it difficult to extrapolate statistics from other regions.

In addition, most Latin American dermatologists are located in big cities, which leaves wide geographic areas with few or no specialists.^66^ Publications that assess the impact of socioeconomic factors on the management, progression, and survival of MCC have demonstrated lower survival in low-income cities with a low density of dermatology specialists.^67,68^ A better understanding of the disease by all health care providers, especially clinicians, would likely increase the number of cases diagnosed. Thus, more and better continuing medical education about skin cancer is needed.

All patients with MCC should be offered a range of therapeutic options and recommendations made according to the most likely best outcome. Nevertheless, it is up to the patient to decide his or her therapy of choice. A multidisciplinary team of health care professionals is needed to provide optimum care for patients with MCC.

Many reports from Latin America describe delays in timely diagnosis and medical assessment as a result of barriers associated with low socioeconomic conditions.^69^ In most case reports, there is no mention of imaging studies performed, and staging is usually made on the basis of routine X-rays, which suggests limited access to other imaging methods, such as CT scan, magnetic resonance imaging, or PET-CT.^70-72^ Thus, substantially more precise epidemiologic information is required in Latin America to make data-driven policies that will improve health care and lead to increased survival of patients with MCC.

To provide the best chance to cure patients with MCC, it is essential that everyone have access to a primary care physician. As most patients present initially with an asymptomatic lesion, the possibility of a physician consultation with a follow-up referral to a dermatologist is the best way to achieve an early diagnosis. Prompt surgery should be the next step.

An important problem in Latin America is related to the surgical treatment of MCC. There is both an insufficient number of trained surgeons in the region and a lack of access to adequate nuclear medicine facilities to support SLNB.

Recognizing the importance of radiotherapy in treating MCC, data suggest that the region lacks a sufficient number of radiotherapy units.^73,74^ Another issue, occurring in all of Latin America, is the distribution of radiotherapy services. Most radiotherapy units are located disproportionately in large cities, thus leaving many large geographic areas underserved.

As a result of financial limitations, many people in Latin America do not have access to chemotherapeutic agents and most do not have access to the newer and expensive immuno-oncologic agents. Health policymakers should make a concerted effort to study the cost-effectiveness of oncology treatments. As more and more cancers can be cured or treated effectively, the impact on health care budgets will be substantial and health care financing will grow in importance.

All of these issues—the absence of robust data with which to understand the impact of MCC, the need for more trained surgeons and better nuclear medicine facilities, and the need for better access to immuno-oncologic drugs—are topics of great concern. Government, nongovernmental organizations, and the medical community must partner to address these issues in a cost-effective manner, thereby providing the region’s population with the health care they deserve.
